# Effects of Non-steroidal Anti-inflammatory Drugs (NSAIDs) and Gastroprotective NSAIDs on the Gastrointestinal Tract: A Narrative Review

**DOI:** 10.7759/cureus.37080

**Published:** 2023-04-03

**Authors:** Rohab Sohail, Midhun Mathew, Khushbu K Patel, Srija A Reddy, Zaroon Haider, Mansi Naria, Ayesha Habib, Zain U Abdin, Waleed Razzaq Chaudhry, Anum Akbar

**Affiliations:** 1 Internal Medicine, Quaid-e-Azam Medical College, Bahawalpur, PAK; 2 Department of Internal Medicine, Pennsylvania Hospital, Philadelphia, USA; 3 Internal Medicine, Index Medical College Hospital & Research Center, Indore, IND; 4 Internal Medicine, Malla Reddy Institute of Medical Sciences, Hyderabad, IND; 5 Internal Medicine, Combined Military Hospital (CMH) Lahore Medical College and Institute of Dentistry, Lahore, PAK; 6 Internal Medicine, American University of Barbados, Bridgetown, BRB; 7 Internal Medicine, Punjab Medical College, Faisalabad, PAK; 8 Medicine, District Head Quarter Hospital, Faisalabad, PAK; 9 Internal Medicine, Services Institute of Medical Sciences (SIMS), Lahore, PAK; 10 Department of Pediatrics, University of Nebraska Medical Center, Omaha, USA

**Keywords:** no-nsaids, dual cox/lox, gastroprotective nsaids, prostaglandins, cox enzyme, nsaids

## Abstract

Non-steroidal anti-inflammatory drugs (NSAIDs) are commonly used for their anti-inflammatory, antipyretic, and analgesic properties. However, their use is often associated with gastrointestinal tract (GIT) side effects due to the inhibition of both cyclooxygenase (COX)-1 and COX-2 enzymes, leading to a decrease in gastroprotective prostaglandins (PG). To minimize these adverse effects, various approaches have been explored, including selective COX-2 inhibitors, NO-NSAIDs (nitric oxide-releasing NSAIDs), and dual COX/LOX (lipoxygenase) NSAIDs. However, the effects of these gastroprotective NSAIDs on the GIT and their efficacy remains uncertain. This review aims to provide an overview of the current understanding of the effects of traditional NSAIDs and gastroprotective NSAIDs on GIT. We discuss the underlying mechanisms of GIT damage caused by NSAIDs, including mucosal injury, ulceration, and bleeding, and the potential of gastroprotective NSAIDs to mitigate these effects. We also summarize recent studies on the efficacy and safety of various gastroprotective NSAIDs and highlight the limitations and challenges of these approaches. The review concludes with recommendations for future research in this field.

## Introduction and background

Nonsteroidal anti-inflammatory drugs (NSAIDs) constitute approximately 5-10% of all prescribed medications worldwide as antipyretic, anti-inflammatory, and analgesic agents. It is estimated that 30 million individuals use NSAIDs daily [1]. In general practice, NSAID usage among patients aged 65 years and above is as high as 96% [2]. Over a 12-month period, at least one NSAID prescription was filled by 7.3% of elderly patients aged over 60 years [3].

In 1860, the Kolbe Company in Germany began mass production of salicylic acid in its chemical form. Bayer later introduced acetylsalicylic acid (aspirin) in powder form in 1899, followed by tablet form, making it a more palatable option for consumers [4]. In 1960, John Vane identified the mechanism of action of NSAIDs which is to inhibit the activity of an important enzyme involved in prostaglandin synthesis known as the cyclooxygenase enzyme (COX) in in vitro settings [5]. COX is present in two distinct forms, COX-1 and COX-2. COX-1 serves vital physiological functions in the body, such as the release of prostacyclin from endothelial cells, which has anti-thrombogenic properties, plays a role in maintaining renal function, and acts as a cytoprotective agent in the gastrointestinal mucosa [4]. Conversely, COX-2 is an inducible isoform of the COX enzyme discovered by Needleman, Simmons, and Herschman's team in the early 1990s, which is induced by inflammatory stimuli and cytokines [6-8]. This finding suggested a theory that the anti-inflammatory effects of NSAIDs are due to their inhibition of COX-2, while their adverse gastrointestinal side effects are due to the inhibition of the COX-1 enzyme [4].

Traditional nonsteroidal anti-inflammatory drugs (tNSAIDs) have been shown to inhibit both isoforms of the COX enzyme, resulting in reduced production of gastroprotective prostaglandins via the COX-1 pathway and increased risk of adverse gastrointestinal (GI) side effects [9]. Studies have demonstrated that the incidence of NSAID-induced GI toxicity is similar for males and females, highlighting the need for the development of gastroprotective NSAIDs that can selectively inhibit the production of inflammatory prostaglandins while sparing the COX-1-mediated production of protective prostaglandins [10]. Such gastroprotective NSAIDs may provide an effective means of reducing the GI toxicity associated with tNSAIDs and improving their overall safety profile, particularly in elderly populations who are at increased risk for NSAID-induced GI toxicity [11].

Various strategies have been employed to develop alternative medications that possess similar therapeutic efficacy to tNSAIDs (traditional NSAIDs), but with fewer gastrointestinal tract (GIT) side effects. Some of these strategies include the development of selective COX-2 inhibitors, nitric oxide (NO)-containing NSAIDs, and dual lipoxygenase (LOX)/COX NSAIDs [12,13]. These approaches were aimed at reducing the adverse effects associated with tNSAIDs while maintaining their beneficial effects in the management of pain and inflammation. The development of these alternative medications has been driven by the need to improve patient outcomes and reduce the burden of adverse events associated with the use of tNSAIDs.

Selective COX-2 inhibitors are a subclass of non-steroidal anti-inflammatory drugs (NSAIDs) that are designed to target the enzyme cyclooxygenase-2 (COX-2), which is responsible for inflammation and pain. Unlike tNSAIDs, which inhibit both COX-1 and COX-2, selective COX-2 inhibitors spare COX-1, which is important for the production of gastric mucosal prostaglandins that help maintain the integrity of the stomach lining [14]. In addition, the modification of NSAIDs with nitric oxide (NO) donors has led to the development of NO-containing NSAIDs, which have been found to be gastroprotective in animal studies [15]. The vasodilatory effects of NO lead to enhanced GI mucosal healing, which can be beneficial in preventing GI side effects. Dual COX/5-LOX inhibitors are another class of non-classical NSAIDs that have been shown to have reduced GI side effects [16]. By inhibiting both lipoxygenase and cyclooxygenase, they can reduce the production of leukotrienes, which can cause adverse effects on the GI tract. This contrasts with tNSAIDs that only inhibit cyclooxygenase and therefore enhance leukotriene production.

The objective of this narrative review article is to systematically gather and critically assess relevant research regarding NSAIDs and their impact on the gastrointestinal tract, with a specific emphasis on gastroprotective NSAIDs such as selective COX-2 inhibitors, NO-donor NSAIDs, and dual COX/5-LOX NSAIDs. The aim is to provide a comprehensive overview of current literature, highlighting any areas of controversy or discrepancies in research findings, and ultimately to provide the medical community with an informative and thorough review of the detrimental effects of NSAIDs on the GIT and alternative strategies for mitigating these effects through the use of gastroprotective NSAIDs.

## Review

Mechanism of action and indications of NSAIDs

Mechanism of Action of NSAIDs

**Figure 1:**
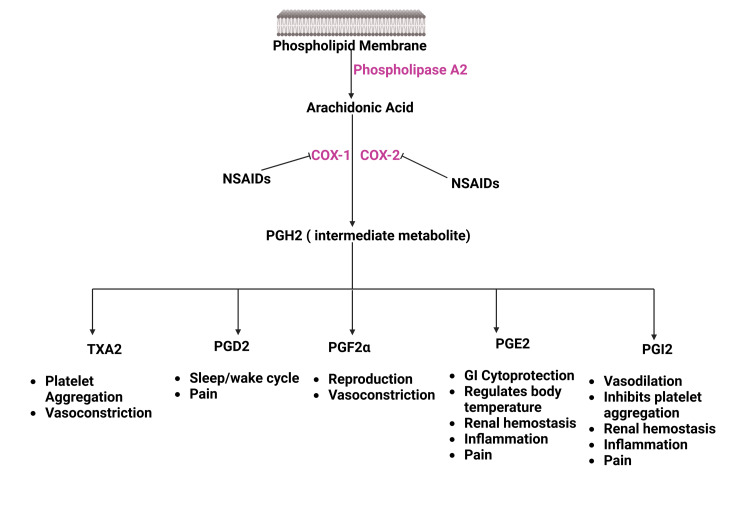
Mechanism of Action of NSAIDs NSAIDs: Non-steroidal anti-inflammatory drugs; COX: Cyclooxygenase; PGH2: Prostaglandin H2; TXA2: Thromboxane A2; PGD2: Prostaglandin D2; PGF2α: Prostaglandin F 2α; PGE2: Prostaglandin E2; PGI2: Prostacyclin Source: Reference no. [19] (Created with bioRender.com)

The fundamental mechanism of NSAIDs is to inhibit COX enzymes. Both isoforms of COX enzyme act on membrane phospholipid known as arachidonic acid to produce different prostaglandins that perform various physiological functions in the body (Figure 1) [17-19].

Indications of NSAIDs

NSAIDs are widely used as anti-analgesic, antipyretic, and anti-inflammatory agents [20]. They are available both over the counter and through medical prescription and are some of the mostly commonly prescribed and utilized medications worldwide [21,22].

NSAIDs for pain: Dysmenorrhea is a common condition among women, characterized by cramping abdominal pain and high levels of prostaglandins (PGs) which are known to cause such pain [23,24]. NSAIDs are commonly used to treat dysmenorrhea by inhibiting PG production through the inhibition of COX action. However, there is insufficient evidence to determine which NSAID is the safest and most effective for this purpose [25].

For acute mild to moderate pain, first-line treatment options include acetaminophen and NSAIDs, while topical NSAIDs are recommended for non-low back musculoskeletal injuries [26]. In cases of severe or refractory acute pain, medications that work on opioid and monoamine receptors or acetaminophen/opioid or NSAID/opioid combinations may be used [27]. However, NSAIDs are not effective for the management of neuropathic pain [28].

Migraine/headache is associated with the sensitization of peripheral nociceptors and the release of neuropeptides leading to neurogenic inflammation and pain. NSAIDs can effectively treat migraines by blocking the production of PG within the central nervous system and their parenteral administration is beneficial for emergency room situations and severe attacks that do not respond to oral treatments [29]. Among NSAIDs, ibuprofen, naproxen sodium, acetylsalicylic acid, and diclofenac potassium have been shown to be effective for the abortive treatment of migraines, while tolfenamic acid, piroxicam, and keterolac may also be used [30].

Oral and topical NSAIDs have been found to effectively reduce pain and swelling associated with sprains and soft tissue injuries [31]. Topical NSAIDs are preferred in patients aged ≥ 75 years due to their similar efficacy to oral medications and reduced risk of systemic adverse effects [32]. Patients using the topical ibuprofen cream had a significant reduction in pain scores over the first 48 h of treatment [33]. Racemic ibuprofen has been used for the management of spondylitis, osteoarthritis, rheumatoid arthritis, and soft tissue disorders [34]. Diclofenac, ibuprofen, tolmetin, and naproxen are equally effective in controlling joint symptoms, while etoricoxib and diclofenac are the most effective oral NSAIDs for pain and function in patients with osteoarthritis [35,36].

For dental pain, ibuprofen and other NSAIDs are commonly used by dental practitioners for acute and chronic dental and orofacial pain [34]. Ibuprofen 400mg provides effective analgesia for the control of postoperative pain after dental surgery in adults, and a liquid gel preparation of ibuprofen 400mg provides faster relief and superior overall efficacy in post-surgical dental pain [37].

NSAIDs for inflammation: NSAIDs are frequently employed for their anti-inflammatory properties in a range of conditions, including osteoarthritis, rheumatoid arthritis, ankylosing spondylitis, bursitis, gouty arthritis, polyarticular juvenile arthritis, tendonitis, tenosynovitis, and other rheumatologic diseases [38]. While some NSAIDs, such as naproxen, have been approved by the FDA for the treatment of inflammatory arthropathies like rheumatoid arthritis and ankylosing spondylitis, they do not alter the disease course or prevent joint and soft tissue destruction that are common sequelae of these conditions. Therefore, disease-modifying anti-rheumatic drugs (DMARDs) have become the first-line treatment for inflammatory arthropathies, with NSAIDs like naproxen utilized as adjunctive therapy [39].

NSAIDs for infection: NSAIDs have been widely used in the management of various respiratory infections, including COVID-19, community-acquired pneumonia (CAP), bacterial infections, and influenza [40,41]. However, their use needs to be balanced with the risks and benefits, as they have diverse structural and pharmacodynamic profiles but similar modes of action. Studies have shown that ibuprofen, a commonly used NSAID, might exacerbate the condition of patients with COVID-19, while some guidelines have recommended its use [42]. In CAP, NSAIDs have not been found to have much of an effect on the reduction of symptoms or duration of acute respiratory infections rather studies suggest that they may worsen the CAP [43]. NSAIDs such as aspirin, ibuprofen, celecoxib, carprofen, bromfenac, and vedaprofen have also been shown to exhibit antibacterial activity, with aspirin inhibiting the growth of *Klebsiella pneumoniae* and *Helicobacter pylori*, and diclofenac possessing antibacterial activity against *Escherichia coli*, *Listeria monocytogenes*, and *Mycobacterium* spp. Ibuprofen and aspirin have also shown antibacterial activity against strains of *Staphylococcus aureus*, which could potentially be used as adjuvants in combating multidrug-resistant MRSA [44]. In influenza, studies have shown that ibuprofen and naproxen have positive effects in controlling cold symptoms and do not cause serious side effects, while the combination of clarithromycin, naproxen, and oseltamivir leads to a decrease in mortality rate and duration of hospitalization in patients with pneumonia caused by influenza [45]. Overall, the use of NSAIDs in respiratory infections requires careful consideration of their potential risks and benefits, as well as their antibacterial activity, to optimize patient outcomes.

NSAIDs for primary prevention: NSAIDs are recommended for primary prevention of cardiovascular disease, especially for those aged 50-59 years. However, caution is needed due to the increased risk of gastrointestinal bleeding, and further studies are required to determine the risk and benefit balance in this population [46].

NSAIDs for off-label Use: NSAIDs for off-label indications has been extensively studied. Ibuprofen is commonly used for the treatment of acute gout flares and pericarditis, although this is not an FDA-approved indication [47]. Celecoxib is increasingly being used in hospital protocols as part of a multimodal perioperative pain management regimen [48]. Indomethacin is used for the treatment of aphthous stomatitis, plantar fasciitis, back pain, and preterm labor, and it has shown promise in the treatment of headache disorders, colorectal cancer, neuropathic pain, and fibromyalgia [38]. While there is limited evidence supporting the use of NSAIDs in some of these off-label indications, they remain a common therapeutic option for many patients. Further research is required to establish their efficacy in these indications fully. These findings may be useful for clinicians considering off-label use of NSAIDs for their patients.

Effects of NSAIDs on gastrointestinal tract

**Figure 2:**
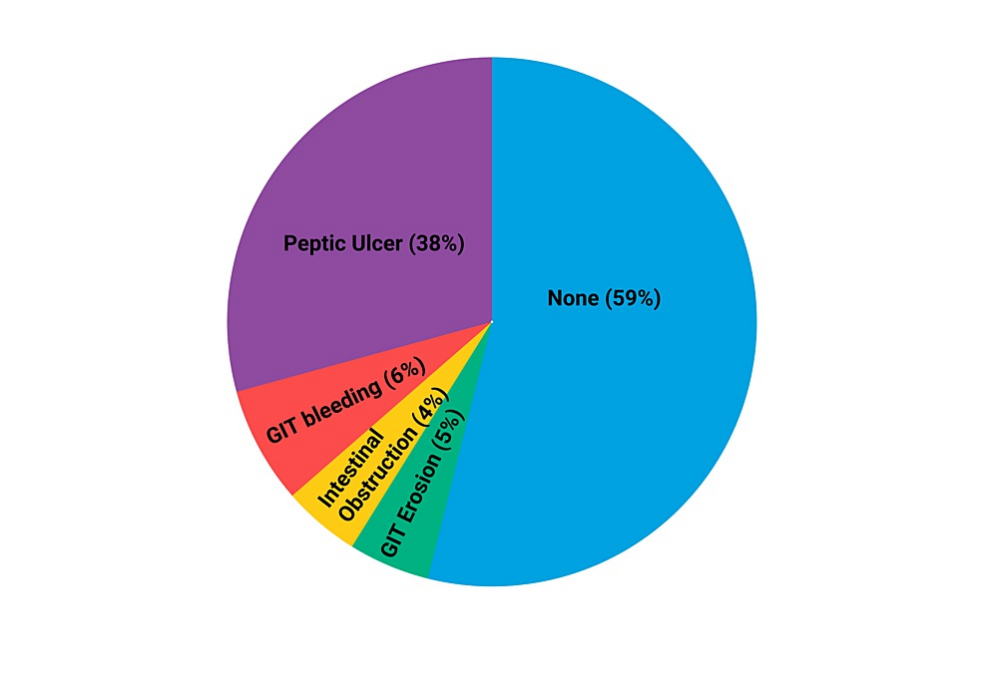
NSAIDs induced GI side effects NSAIDs: Nonsteroidal anti-inflammatory drugs; GI: Gastrointestinal Source: Reference no. [50] (Created with bioRender.com)

NSAIDs have a global prevalence in usage; however, extensive research has highlighted their detrimental effects on various bodily systems, including the gastrointestinal, cardiovascular, renal, biliary, and hematological systems [49]. Specifically, NSAIDs are recognized as being harmful to the gastrointestinal tract, where the upper gastrointestinal tract is subjected to a significant burden of side effects (Figure 2) [50]. 

There are several risk factors that increase the risk of NSAIDs induced GIT side effects including old age (>60 years) comorbidities (cardiovascular, hepatic, or renal disease), concomitant use of corticosteroid therapy and more than one NSAID, and smoking, etc. [51-53]. The use of gastroprotective slow-release preparations shifted the disease burden to lower GIT and has set the way for several researchers to find the side effects of NSAIDs in the small and large intestines [54].

Effect of NSAIDs on Upper Gastrointestinal Tract

Over the decades, the effects of NSAIDs on the upper GIT have been the prime focus of researchers, which has led to the availability of vast data suggesting the mechanism through which NSAIDs induces GIT side effects. Symptoms can range from mild (such as dyspepsia, gastroesophageal reflux, nausea, and abdominal pain) to severe (GIT bleeding, perforations, and gastritis) [49,52,55]. Dyspepsia (chronic indigestion that presents as stomach fullness, bloating, and upper abdominal pain during or immediately following food intake) is not only the most common side effect of NSAIDs but also the leading cause of premature discontinuation of the drug [49,52,56]. Although there are several other causes of dyspepsia besides NSAIDs, it is crucial to determine the association of dyspepsia with NSAID use as 10% of patients stop using NSAIDs because of associated dyspepsia [51,54]. Its diagnosis is made clinically, and no structural lesion is found on endoscopy [57].

NSAIDs use is also associated with esophagitis and affected patients present with complaints of dysphagia, odynophagia, or abdominal pain [58]. Endoscopy showing damage to the mucosal lining of the esophagus and pill components adherent to the esophageal lining confirms the diagnosis [59].

People taking NSAIDs are more likely to experience gastroesophageal reflux, presenting with heartburn, cough, metallic taste in the mouth, and regurgitation [56]. While studying the mechanism of action of NSAIDs, it was concluded that NSAIDs are renowned for negatively altering the tone of the gastroesophageal sphincter and peristalsis in the esophagus [60]. A relaxed sphincter and reduced esophageal motility led to regurgitation of gastric contacts into the esophagus, chronic exposure of mucosal lining of the esophagus to gastric contents results in barrette esophagus, and esophageal stricture [61,62]. On the contrary another study showed that NSAIDs use lowers the risk of developing esophageal adenocarcinoma, making it a protective factor [63-65].

**Figure 3:**
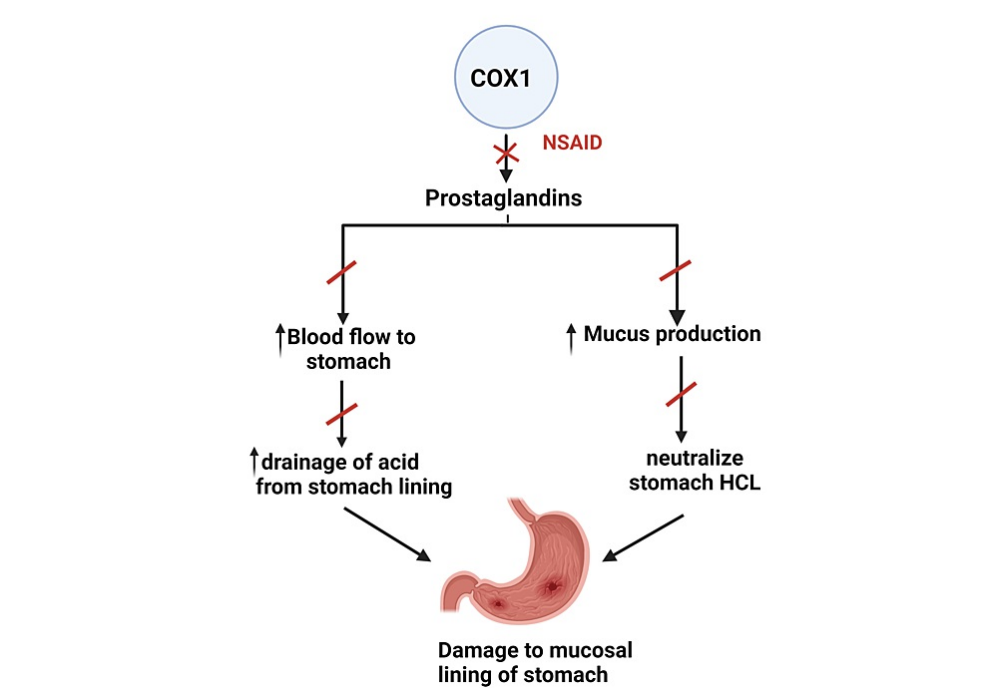
COX-1 inhibition induced stomach ulcer COX: Cyclooxygenase Source: Reference no. [52] (Created with bioRender.com)

Traditional NSAIDs inhibit COX-1, leading to the inhibition of prostaglandin E2(PGE2) synthesis [57,60,66,67]. Since PG have gastroprotective action by increasing blood flow to stomach mucosa and augmenting the synthesis of mucus that lines and protects the stomach wall from hydrochloric acid (HCL) induced damage which is considered as a primary agent that harms the stomach mucosal lining [60]. Acid-induced mucosal damage is categorized either by the partial loss of mucosa (erosion) or the full-thickness loss of mucosa with exposure to underlying submucosa (ulcer) (Figure 3) [51,52,67]. 

The diagnosis is made by visualization of erosion or ulceration via endoscopy [52,68]. Patients with NSAIDs induced ulcers are prone to develop ulcer-related complications that involve perforation (presents with signs of peritonitis), hemorrhage (commonly in ulcers along the lesser curvature that involve the left gastric artery), and obstruction (gastric outlet obstruction due to stricture formation [49,52,57]. The possible mechanism for complications such as hemorrhage due to NSAIDs induced ulcer is that NSAIDs inhibit thromboxane synthesis in platelets that blocks platelet adhesion and aggregation and thus lead to hemorrhage [61]. Ulcer-related complications are the leading cause of hospitalization and death among chronic NSAIDs users [57,69] Prolonged use of NSAIDs leads to chronic exposure of the mucosal lining to toxic agents, resulting in gastritis (inflammation of the gastric lining) [68]. Interestingly, NSAIDs by inhibition of COX protects against intestinal type gastric carcinoma, this protective effect is also seen in esophageal and colonic carcinoma [63,64].

Effects of NSAIDs on the Small Intestine

While a significant number of patients who use NSAIDs over a long period of time do not experience any GIT symptoms, a minority of them may exhibit symptoms due to pre-existing intestinal barrier dysfunction or ulceration [70].

The inclusion criteria for NSAID-induced small intestinal injury include; 1) history of NSAID use 2) Endoscopic findings of erosion, ulcer, or diaphragm-like stricture with non-specific tissue biopsy findings 3) improvement in signs and symptoms or endoscopic findings after cessation of NSAIDs [71].

According to a previously proposed 3-hit hypothesis, NSAID-induced enteropathy involves direct damage to mucosal cell membrane phospholipids and subsequent mitochondrial injury, leading to decreased energy synthesis, calcium efflux, and free radical generation. This disrupts intercellular junctions, increases mucosal permeability, and allows intraluminal contents to invade cells, activate inflammatory pathways, and result in clinical manifestations such as erosions, bleeding, ulceration, and protein loss [72]. Furthermore, recent studies have also shown that NSAIDs cause significant damage to the small intestine due to the suppression of gastric acid, thereby inhibiting the action of gastric acid on the gram-negative bacteria which causes ulceration in the small intestine [70].

The injuries induced by NSAIDs in the small intestine were initially studied by enterostomy and later on via capsule endoscopy [70,71,73]. Some studies also used fecal calprotectin to assess the small intestine damage from NSAID [70,74]. The enteroscopic features of NSAID-induced enteropathy mainly included red spots, erosion, and morphologic ulcerations such as round/longitudinal/annular ulcers, and linear ulcers/scar and diaphragm stricture (rarely detected) [71]. The most common signs of NSAID-induced enteropathy are occult GI bleeding (blood loss ranges between 1-10ml/day) with resultant iron deficiency anemia and protein loss resulting in hypoalbuminemia [75]. While the most common complications are bleeding, perforation and obstruction [72]. The diaphragm stricture that develops in the intestine is pathognomy of NSAID use which usually comprises 2-4 mm septate in the mid-small intestine thereby reducing the gut lumen to a pinhole [73].

Effect of NSAIDs on the Large Intestine

The adverse effects of NSAIDs on the upper GIT and small intestine have been well established but the effect on the large intestine termed NSAID colopathy is not well recognized [76]. Although slow release formulation have reduced risks of NSAID-induced side effects on the upper GIT such as gastritis, esophagitis, and ulcer, it does not prevent the harmful effects of NSAIDs on the large intestine particularly the colon [76]. NSAIDs has shown to cause erosion, ulcer, diaphragm like stricture in large intestine [77,78] The long term use of NSAIDs can lead to the development of diaphragm like stricture (from fibrous proliferation) which eventually require therapeutic interventions like balloon dilatation or even segmental colectomy [77]. Histology of NSAIDs induced colitis is characterized by patchy inflammation with lymphoplasmocytic and neutrophil cells and is associated with slight crypt disarray and focal erosion [79]. The endoscopic appearance of sharply demarcated ulceration at the crest of a mucosal web with adjacent normal mucosa is known to be characteristic of NSAID-induced colitis, especially when the ulcers are in the right colon [80]. Interestingly, animal studies showed that NSAIDs might protect against large intestine cancer via modulating cellular proliferation [78].

Gastroprotective NSAIDs

NSAIDs are commonly used in various populations, but their traditional formulations are associated with upper and lower gastrointestinal (GIT) side effects [49]. While slow-release formulations reduce the upper GIT side effects, the lower GIT side effects still pose a concern. To mitigate these side effects, strategies such as using COX-2 selective NSAIDs, NO-releasing NSAIDs, and dual COX/LOX inhibiting drugs have been employed, which have similar therapeutic efficacy to tNSAIDs but with a lower risk of GIT side effect [54].

Selective COX-2 Inhibitors

**Figure 4:**
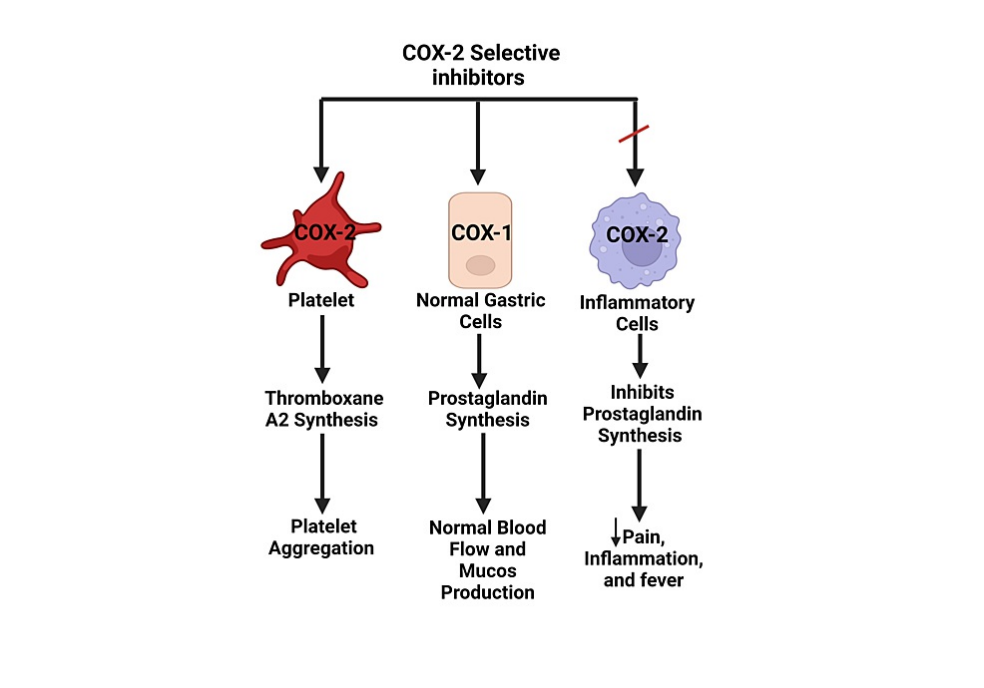
Mechanism of Action of Selective COX-2 inhibitors COX: Cyclooxygenase Source: Reference no. [49] (Created with bioRender.com)

Research on steroids has revealed that their anti-inflammatory properties are achieved through the inhibition of leukotriene enzymes as well as a newly discovered class of enzymes that bear similarities to COX enzymes, which has been designated COX-2 [81]. This facilitated the development of the first-generation COX-2 selective NSAIDs, including celecoxib and rofecoxib, which were approved by the FDA in 1998 and 1999 [82]. However, rofecoxib was withdrawn from the market in 2004 due to its association with cardiovascular events, as revealed by subsequent studies [83]. Selective COX-2 inhibitors are known to possess similar anti-inflammatory and analgesic properties as tNSAIDs [84]. COX-1 is expressed in naturally occurring cells throughout the body, while COX-2 is specific to cells involved in inflammation [67]. tNSAIDs block PG synthesis in both inflammatory and normal cells of the GIT, while COX-2 selective NSAIDs primarily inhibit PG synthesis involved in pain and inflammation, leaving PG synthesis in normal cells intact (Figure 4) [49].

Furthermore, the use of tNSAIDs inhibit thromboxane A2 and prevent platelet aggregation carries an increased risk of ulcer-associated bleeding. However, the use of COX-2 selective inhibitors does not exhibit such an effect. Several randomized control trials, meta-analyses, and systematic reviews have demonstrated the GIT protective effect of COX-2 selective NSAIDs, including the CLASS, VIGOR, TARGET, and SUCCESS-1 studies [85-88]. Celecoxib was found to have less GIT toxicity when compared to tNSAIDs in multiple meta-analyses and clinical trials [89].

NO-NSAIDs

The incorporation of nitroxybutyl or nitrosothiol moiety into conventional NSAIDs by a short-chain ester linkage has resulted in the development of a new class of drugs known as nitric oxide releasing NSAIDs (NO-NSAID) [90]. The concept of NO-NSAID was introduced in the 1990s by John Wallace and colleagues as a way of reducing the gastrointestinal toxicity associated with conventional NSAIDs [91]. In addition to decreasing gastric toxicity, NO-NSAIDs have also been found to have cardiovascular and renal benefits and enhance anti-inflammatory effects [92]. Long-term use of NSAIDs has been associated with a decreased incidence of colon cancer, and NO-NSAIDs have been found to have better antiproliferative activity and prevent tumor growth compared to conventional NSAIDs [93]. They also hold promise as a safer alternative for chemoprevention due to their gastroprotective effects.

NO-NSAIDs retain the anti-inflammatory and antipyretic properties of parent NSAIDs while also providing gastroprotective benefits by releasing NO. This is achieved by vasodilating the gastric vasculature, inhibiting neutrophil adhesion to vascular endothelium and inactivating caspases [90,93]. While NO-NSAIDs are not currently approved for use in the US market, several drugs are undergoing clinical trials and more clinical data is needed for approval [94].

Dual LOX/COX Inhibitors

COX-2 selective NSAIDs were initially introduced to alleviate the adverse effects of traditional NSAIDs, but their use still results in undesired consequences [95]. Consequently, the development of safer NSAIDs with dual COX and LOX inhibition became necessary to minimize the negative effects of NSAIDs on the gastrointestinal mucosa [96]. Both COX-1 and COX-2 utilize arachidonic acid as a substrate, which generates leukotrienes that play a significant role in inflammatory processes [96]. Studies have shown that COX inhibitors alone can shift the breakdown of arachidonic acid toward leukotriene production, thereby exacerbating inflammation, particularly with LTB4 [97]. Dual COX-2/LOX inhibitors are a promising new class of drugs that may provide better gastroprotection than tNSAIDs by inhibiting both the COX and LOX pathways, thereby reducing the adverse effects of NSAIDs on the GIT mucosa [97]. Although there are no approved dual COX/LOX drugs but clinical trials have shown to be very promising, dual COX-2/LOX inhibitors have also demonstrated an ability to inhibit cancer cell proliferation, with tepoxalin and licofelone being two drugs under clinical trials that have displayed anti-inflammatory effects and are less ulcerogenic than indomethacin. Finally, while celecoxib has been shown to increase gastric mucosal damage in aspirin-treated rats, licofelone does not have this effect [98].

Discussion

Non-steroidal anti-inflammatory drugs (NSAIDs) are widely prescribed medications that have proven to be effective in treating pain, inflammation, and fever [99,100]. These drugs exert their pharmacological effects by COXs enzymes, which are the rate-determining enzymes for the synthesis of prostaglandins and other prostanoids, such as thromboxanes [101]. tNSAIDs inhibit both COX-1 and COX-2. COX-2 is primarily involved in prostaglandin-mediated pain and inflammation, whereas COX-1 has a housekeeping role in the protection of gastric mucosa and platelet hemostasis [102].

NSAIDs are commonly used for the management of conditions such as muscle pain, dysmenorrhea, arthritic conditions, pyrexia, gout, migraines, and as opioid-sparing agents in certain acute trauma cases [103]. In addition to their analgesic, anti-inflammatory, and antipyretic properties, NSAIDs have been shown to offer protection against a range of critical disorders, including cancer and heart attacks [41].

Examples of FDA-approved NSAIDs available in the market include diclofenac, etodolac, fenoprofen, flurbiprofen, ibuprofen, indomethacin, and ketorolac [9]. These drugs have demonstrated their efficacy and safety in numerous clinical studies, and they remain one of the most commonly prescribed medications worldwide.

It is worth noting that NSAIDs are not without potential side effects, including gastrointestinal bleeding, renal dysfunction, and cardiovascular events. Physicians should exercise caution when prescribing NSAIDs, particularly in patients with pre-existing conditions such as hypertension, renal disease, or a history of peptic ulcer disease. The cardiovascular, renal, hepatobiliary, hematological, and gastrointestinal tracts are the most commonly affected organs, with the esophagus and stomach bearing the primary burden [49,50]. NSAIDs can cause esophagitis by their direct irritating effect on the esophageal epithelium and can also reduce the motility of the lower esophageal sphincter and stomach, leading to gastroesophageal reflux and dyspepsia [52]. NSAIDs can irritate the gastric epithelium lining, resulting in gastritis, and increase the risk of erosion and ulcer formation by inhibiting mucus production by the stomach's goblet cells and decreasing the acid washout from the gastric epithelium [57,60,66,67]. Moreover, NSAIDs can decrease thromboxane synthesis, which leads to bleeding in the gastric epithelium [61]. However, NSAIDs have a protective role against esophageal and stomach carcinomas [63-65].

NSAID enteropathy is as frequent and severe as NSAID gastropathy, and recent advances in diagnostic devices have shed light on this issue. The most commonly affected areas are the distal small bowel, terminal ileum, and ileocecal junction, and imaging studies have revealed erosions, bleeding, ulceration, stricture formation, and perforation [104]. Anemia and hyperproteinemia are the most common clinical findings. The 3 hit hypothesis explains the mechanism of NSAID-induced small-bowel injury in detail [105].

NSAID colopathy is being increasingly reported in the literature, but limited data are available compared to NSAID gastropathy and NSAID enteropathy. The diagnosis of NSAID colopathy can be challenging due to the varied nature of symptoms, which may resemble those of inflammatory bowel disease and malignancy sometimes. Moreover, it remains unclear whether the use of NSAIDs triggers or exacerbates inflammatory bowel disease, and if there exists any correlation between inflammatory bowel disease and NSAID usage [106]. NSAID colopathy is suspected if endoscopy shows diaphragm-like strictures associated with ulceration [107]. The patient presentation usually includes abdominal pain, anemia, rectal bleeding, diarrhea, obstruction, and perforation. The prevalence of NSAID colopathy is slowly rising due to the use of enteric-coated or slow-release NSAIDs to protect the upper gastrointestinal tract, enabling these drugs to reach the colon disintegrated [108]. NSAIDs have detrimental as well as beneficial effects on the large intestine, including protection against colorectal cancer and regression in the size of colonic polyps. Withdrawal of NSAIDs usually alleviates the symptoms of NSAID-induced colopathy [109]. Withdrawal of the offending agent is always not practical, and various other strategies implemented to reduce small bowel damage include slow-release/enteric-coated formulations, and using selective COX-2 inhibitors [110]. Experimental prodrugs like NO-NSAIDs and COX/LOX inhibitors have shown promising data in clinical trials. Overall, the use of NSAIDs requires careful consideration of the potential risks and benefits, and further research is needed to develop safer alternatives or strategies to minimize the harmful effects of these drugs.

In 1998, the FDA approved COX-2 selective inhibitors as an alternative to tNSAIDs to prevent the GIT side effects [111]. Currently, COX-2 inhibitors are used to treat inflammatory joint diseases and pain [84,112,113]. However, the withdrawal of rofecoxib from the US market due to its association with an increased risk of cardiovascular events has raised concerns about the safety of other COX-2 inhibitors [83]. Therefore, further studies are required to determine whether other COX-2 inhibitors pose a similar risk to their users.

Nitric oxide releasing NSAIDs (NO-NSAIDs) are a new class of "Safe-NSAIDs" that are currently in the clinical trial phase [114]. In animal studies, they have exhibited markedly reduced gastrointestinal toxicity while retaining the anti-inflammatory and antipyretic activity of the parent NSAID [90]. These compounds are made by attaching a nitric oxide releasing moiety to the parent NSAID via an ester linkage, which causes vasodilation in the gastrointestinal tract, thus maintaining mucosal integrity. In addition, NO-NSAIDs have shown potential benefits in colon cancer by inhibiting cancer cell growth [115]. Overall, NO-NSAIDs have the potential to be a safer alternative to traditional NSAIDs and COX-2 inhibitors for the treatment of inflammatory conditions.

Finally, exploring the potential benefits of dual COX/LOX inhibitors is another strategy for mitigating the GIT side effects of NSAIDs. Research has demonstrated that these inhibitors offer gastroprotective effects and possess the ability to inhibit the proliferation of cancer cells [98]. Thus, they hold promise as a viable alternative to tNSAIDs.

While our review article provides a thorough examination of the available literature on NSAIDs, gastroprotective NSAIDs such as COX-2 Inhibitors, NO-NSAIDs, and dual COX/LOX inhibitors and their effects on the GIT, it is important to acknowledge several limitations that may have affected our findings. One potential limitation is that our search was limited to articles published in the English language, which may have excluded relevant studies published in other languages. Secondly, we did not have access to unpublished data or ongoing clinical trials, which may have provided further insight into the efficacy of gastroprotective NSAIDs. Lastly, studies such as metanalysis or systemic analysis need to be done to compare the efficacy, safety, and toxicity of these drugs with tNSAIDs. Despite these limitations, we believe that our review provides a comprehensive summary of the current literature on gastroprotective NSAIDs and can serve as a valuable resource for researchers and healthcare professionals in this field.

## Conclusions

In conclusion, NSAIDs are frequently utilized due to their beneficial analgesic and anti-inflammatory effects. Nevertheless, their administration is associated with various gastrointestinal adverse effects, especially in high-risk individuals. Consequently, healthcare professionals should carefully consider the risks and benefits of NSAID therapy for each patient and explore alternative treatments as appropriate. Furthermore, randomized controlled trials are needed to assess and compare the efficacy of gastroprotective NSAIDs with tNSAID. Overall, it is essential to achieve a balance between the potential benefits and harms of NSAID therapy to optimize patient outcomes. This emphasizes the need for clinicians to exercise caution and tailor their approach to each patient when utilizing NSAIDs for pain and inflammation management.
